# Three-Dimensional-Printed Composite Scaffolds Containing Poly-ε-Caprolactone and Strontium-Doped Hydroxyapatite for Osteoporotic Bone Restoration

**DOI:** 10.3390/polym16111511

**Published:** 2024-05-27

**Authors:** Cosmin Iulian Codrea, Daniel Lincu, Vladimir Lucian Ene, Adrian Ionuț Nicoară, Miruna Silvia Stan, Denisa Ficai, Anton Ficai

**Affiliations:** 1 Faculty of Chemical Engineering and Biotechnologies, National University of Science and Technology Politehnica of Bucharest, 060042 Bucharest, Romania; ccodrea@icf.ro (C.I.C.); daniel.lincu1113a@gmail.com (D.L.); adi.nicoara18@gmail.com (A.I.N.); denisaficai@yahoo.ro (D.F.); anton_ficai81@yahoo.com (A.F.); 2 Institute of Physical Chemistry “Ilie Murgulescu” of the Romanian Academy, 060021 Bucharest, Romania; 3 National Research Center for Micro and Nanomaterials, Faculty of Chemical Engineering and Biotechnologies, National University of Science and Technology Politehnica of Bucharest, Splaiul Independentei 313, 060042 Bucharest, Romania; 4 National Centre for Food Safety, National University of Science and Technology Politehnica Bucharest, Splaiul Independentei 313, 060042 Bucharest, Romania; 5 Department of Biochemistry and Molecular Biology, Faculty of Biology, University of Bucharest, Splaiul Independentei 91-95, 050095 Bucharest, Romania; miruna.stan@bio.unibuc.ro; 6 The Academy of Romanian Scientists, Ilfov St. 3, 050044 Bucharest, Romania

**Keywords:** hydroxyapatite nano-powder, co-precipitation, hydrothermal, strontium, poly-ε-caprolactone, 3D printing, bioactivity

## Abstract

A challenge in tissue engineering and the pharmaceutical sector is the development of controlled local release of drugs that raise issues when systemic administration is applied. Strontium is an example of an effective anti-osteoporotic agent, used in treating osteoporosis due to both anti-resorptive and anabolic mechanisms of action. Designing bone scaffolds with a higher capability of promoting bone regeneration is a topical research subject. In this study, we developed composite multi-layer three-dimensional (3D) scaffolds for bone tissue engineering based on nano-hydroxyapatite (HA), Sr-containing nano-hydroxyapatite (SrHA), and poly-ε-caprolactone (PCL) through the material extrusion fabrication technique. Previously obtained HA and SrHA with various Sr content were used for the composite material. The chemical, morphological, and biocompatibility properties of the 3D-printed scaffolds obtained using HA/SrHA and PCL were investigated. The 3D composite scaffolds showed good cytocompatibility and osteogenic potential, which is specifically recommended in applications when faster mineralization is needed, such as osteoporosis treatment.

## 1. Introduction

An increasing number of researchers have turned their attention to making new materials with controllable properties and interactions for bone tissue regeneration. These biomaterials should mimic not only the bone composition and its micro and nanostructure, but also its functions, while taking into account the different needs of each patient [1]. Osteoporotic patients are particularly prone to challenging complications as it is known that healing time and failure rate are increased for them. They require supplementary pharmacological measures to improve bone healing in the short term and overall quality in the long term [2]. Hydroxyapatite (HA) is extensively used as a bone graft material in both orthopedic and dental applications due to its capacity to form strong bonds to natural bone tissue [3]. Its main disadvantages are inherent brittleness [4], low mechanical properties (the relatively low loading capacity), low biodegradation rate, deficiency in osteoinduction, and absence of targeting efficiency as delivery systems [5]. Low fatigue resistance and poor degradability make it improper as artificial bone tissue material directly [6] and it is not used in bone grafting for load-bearing applications nor in its bulk form [4]. The mechanical stiffness characteristic of HA that contributes to its osteogenic bioactivity also makes its surgical implementation challenging, as porous HA cannot be easily shaped and resized by surgeons on demand to better accommodate the defect site [7].

Sr can prompt osteogenesis and inhibit inflammation, while Sr-doped amorphous calcium phosphates (CaP) porous scaffolds improve new bone formation [8], thus attracting considerable interest, especially as Sr-substituted HA. However, ceramic constructs cannot be deformed without failing through fracture. Therefore, CaP-based products have different formulations, such as granules, malleable putties, or injectable cement created with plasticizers, monomers, and water, making them easier to pack into the surgical sites, but even so, suffer from common deficiencies. They are often washed away because of intraoperative bleeding within the defect site, can transfer significant heat because of the chemical curing process and can damage surrounding tissues, and have minimal interconnected porosity, which hinders host–implant integration, vascularization, and patient recovery while also increasing susceptibility to inflammation, infection, and even failure and thus revision surgeries [7].

Polymers support cell attachment, anchorage, and proliferation but in general, are not sufficient for a complete bone reparation due to their lack of osteopromotive ability [9]. Polymer–ceramic composites represent alternative options for bone substitution and regeneration as they are closer to replicating the bone properties and fulfilling all the requirements of artificial bone scaffolds [10]. Integration of ceramics into polymeric matrix determines bone substitute materials to be flexible and strong [4]. As reinforcing material in composite materials, HA can improve the matrix material’s biocompatibility, stimulate bone regeneration, and improve the stiffness, compressive, and bending strengths [4]. Rod and wire-shaped, as well as sheet-shaped particles, are utilized as the mechanical reinforcement component for the preparation of composite materials [5]. Polymer fiber networks provide the organizational framework and spatial constraint for crystal deposition, whereas non-collagenous matrix macromolecules might be involved in the control of nucleation and growth of the mineral phase [11]. Biomaterial scaffolds should have mechanical properties similar to natural bone, namely a stiffness between 17–20 GPa [4].

One of the synthetic biocompatible polymers used for tissue engineering is the aliphatic semi-crystalline polyester, poly-ε-caprolactone (PCL), known alongside poly(lactic-co-glycolic acid) (PLGA) and polylactic acid (PLA) to have good processability and tunable mechanical properties [12]. PCL has the advantage of prolonged degradation rates and U.S. Food and Drug Administration (FDA) approval, but its hydrophobic and nonosteogenic nature decreases cell adhesion and bioactivity when implanted, making it improper for use in pure form [13]. Depending on the proportion of HA nanoparticles used for the composite preparation, the mechanical properties can be controlled for the desired application. The gradual degradation of scaffolds leads to gradual stress stimulation of the bone, necessary for bone regeneration and bone structure remodeling [6]. The relatively low osteoconductive potential of polymers can increase with the addition of HA [9]. Polymer–HA composite scaffolds can be loaded, if necessary, with bioactive factors [6] since the porous HA aggregations with hierarchical architectures have drug loading and controllable release capacity according to the desired kinetic [5,14].

One promising method to properly design biomaterials for the recovery of osteoporotic bone tissue after traumatic injuries is the use of the innovative 3D printing of bone scaffolds method. Due to recent advances in 3D printing technologies, the design and the fabrication of many different scaffold geometries were made possible, with pores of different shapes and dimensions. The scaffold must be designed to trigger the favorable biophysical stimuli, necessary for the formation of new bone tissue. The rate of bone tissue regeneration and the cellular response is influenced, in addition to the scaffold material composition, by the following: (a) the scaffold mechanical behavior, which is a function of the scaffold micro-architecture and of the mechanical properties of the material it is made from; (b) the surface roughness status and the biological/chemical response of the scaffold/tissue interface surfaces to external factors [15].

Composites containing CaP, even in 3D-printed form, have improved stiffness (elastic and compressive moduli) compared to polymeric scaffolds and increased mechanical elasticity or malleability over solely CaP, but a possible issue is that the polymeric component often physically encapsulates the bioactive CaP particles, isolating them from the tissue and mitigating their therapeutic potential [7,16]. Polymer–HA composite scaffolds for bone tissue engineering can mimic the composition and morphology of the bone mineral phase much better than sintered ceramics, through low-crystalline, non-stoichiometric, and nanostructured Ca-deficient HA and polymer matrix [17].

Fused Deposition Modeling (FDM) is a widely and easily used, low-cost additive manufacturing technique [18] that enables the creation of controlled and regular 3D structures in a reproductive manner [9] with the help of computer-aided design (CAD). FDM is an extrusion technique in which the molten polymer leaves the extruder as a liquid and solidifies on the receiving platform [9]. The FDM technique is suitable to build accurate scaffold samples only in the cases where the filament diameter is close to the nozzle diameter [19]. It has technical and medical advantages through its capacity to be rapidly manufactured into small or large, simple or complex, porous constructs via simple, room temperature extrusion-based 3D printing of instantly drying 3D ink [7].

While there are numerous studies regarding the positive effects of the SrHA in bone regeneration [20], and good results were obtained also through electrospinning [21], the controlled design of 3D printing is considered to enhance biological activity and mechanical properties [22]. In this study, by maintaining consistency in scaffold design and 3D printing technology, we examined the effect of different Sr^2+^ concentrations regarding the biological and mechanical properties of the obtained composite scaffolds. Our study provides, coupled with the previous characterization of the nanopowders used in the composite materials [23], a supplementary comparison of the effect different SrHA synthesis methods have on the composite scaffold properties. We chose melt-blending for composite preparation for its ease of use, good reported results [13], and no need for additional solvents. Due to the presence of Sr^2+^, these scaffolds are expected to have an anti-osteoporotic behavior. For this kind of application, the long-term release of active agents is crucial, and through the incorporation of SrHA into the polymer matrix, rather than using just a coating of the scaffolds, a more sustainable approach is achieved. Also, the 70% (wt.) PCL–30% (wt.) HA/SrHA composition of the scaffolds was chosen based on the consistently better reported results that higher HA/SrHA content scaffolds had in similar studies [24,25,26,27]. A more elevated HA/SrHA content is difficult to obtain due to the extrusion method limitations caused by increasing viscosity of the material, an effect cited by other authors as well [26], and lower HA/SrHA content maintains the unsuited physiological slow degradation rate specific to PCL [27,28].

## 2. Materials and Methods

### 2.1. Synthesis of HA and SrHA Powders

HA and SrHA powders were synthesized using the precipitation and hydrothermal methods and thoroughly characterized, as presented in our previous literature report [23]. Molar ratios of Sr/(Ca+Sr) were designed to be 0, 1, 5, 10, 20, and 30%. Samples were referred to as HAPR and HAPR-SrX% for the precipitation method, respectively, HAHT and HAHT-SrX% for the hydrothermal method, where X represents the Sr/(Ca+Sr) molar ratio.

### 2.2. Design and Manufacturing of 3D Composite Scaffolds

Melt-blending method was chosen because previous studies showed a relatively uniform strand diameter of the 3D-printed scaffolds fabricated in this manner, compared to other material preparation techniques, which resulted in scaffolds with less uniformity [13]. Also, the material preparation technique may impact the post-printing properties of the fabricated scaffold. Melt-blended materials demonstrate a slightly greater degree of printability, closely conforming to the CAD model dimensions. Swelling and degradation analysis determined that melt-blended material demonstrated reduced swelling and degradation compared to other material synthesis techniques [13].

Previous research using the FDM technique shows that a high ratio of mineral content can reduce the printability of the composite materials and clogging of the printing head can occur. Also, a higher printing temperature may be needed to print polymer–ceramic composite materials with a high percentage of ceramic [9]. After several iterations, we established the 30% wt. HA/SrHA concentration and the printing temperature of 145 °C, which were associated with good printing quality and reproducibility. We used the 3D printing FDM technique to prepare scaffolds with 0°/90° printing angles. Previous results in the literature showed that similarly designed scaffolds achieved better cell adhesion, proliferation, and differentiation while exhibiting adequate mechanical properties and degradation time [6,7].

Scaffolds were printed from composites containing 70% (wt.) PCL (average Mn = 45,000) pellets purchased from Aldrich, and 30% (wt.) HA/SrHA powder we previously synthesized [23]. PCL was mixed with HA/SrHA through melt-blending. Hence, the PCL pellets were melted in a beaker at 90 °C, while HA/SrHA powder was added to the molten PCL and mixed until homogenous. To avoid the clogging of the needle during the extrusion process, before mixing with the PCL, both HA and SrHA powders were sieved using an analytical sieve with a mesh size of 45 μm. The newly formed printing mixture consisting of PCL-HA/SrHA was left to cool before being cut into small pieces and stored at room temperature.

Roughly cut composite material was placed into a high-temperature stainless-steel cartridge, the temperature was adjusted to 145 °C and kept at this temperature for 30 min to completely melt the materials, and subsequently extruded through a blunt tip needle (0.4 mm inner diameter) of a 3D Bioplotter extrusion system (EnvisionTEC, Gladbeck, Germany). A 3D porous cylindrical model (2.52 mm height, 10 mm diameter) was designed in Bioplotter RP 3.0 software, then the computer-aided design (CAD) model of 8 layers was sliced at 360 μm slicing thickness and uploaded into Visual Machines 2.8.115 Software (EnvisionTEC, Gladbeck, Germany). After an initial optimization process, final printing conditions (temperature, pressure, speed, pre- and post-flow) were set for the scaffolds. Cylindrical specimens were printed continuously, with 0.6 mm between strands, with no offset between layers, at a print speed of 0.3 mm/s and pressure of 5.7 bar. All scaffolds were printed at room temperature.

Control scaffolds were printed from only PCL pellets, maintaining the multi-layer architecture and inner pattern, but we used a temperature of 130 °C, a print speed of 0.5 mm/s, and a pressure of 5 bar, due to the lower viscosity of solely PCL.

### 2.3. Characterization Methods of 3D Composite Scaffolds

#### 2.3.1. General Overview of Characterization Methods

The obtained composite 3D-printed scaffolds were characterized using several methods to evaluate their suitability for bone tissue engineering applications. Characterization methods were selected to obtain a comprehensive understanding of the scaffold’s properties. Thermogravimetric (TG) analysis was used to determine the inorganic/organic content of the composite 3D-printed scaffolds. Scanning Electron Microscopy (SEM) was used to reveal the morpho-structural details of the scaffold surface and cross-section. The analysis of the mechanical properties allows us to evaluate the alignment between the predicted forces exerted on native bone during normal function with the designing, fabrication, and integration of a printed scaffold with the host. Chemical bioactivity evaluation offers insights into the capacity of the scaffolds to interact and integrate successfully into the native bone tissue. Cytotoxicity studies were used to evaluate the scaffold’s biocompatibility and assess its ability to avoid adverse reactions.

#### 2.3.2. Thermogravimetric (TG) Analysis

TG was performed using a Mettler Toledo TGA/SDTA851e instrument under 80 mL/min synthetic air flow, using open ceramic pans. The heating rate was adjusted at 10 °C/min and the temperature range was between 25° C and 800° C.

#### 2.3.3. Scanning Electron Microscopy (SEM) Analysis

SEM measurements were performed using a field emission gun scanning electron microscope (FEG-SEM) Quanta Inspect F50, with a resolution of 1.2 nm. The surface morphologies of the scaffolds as well as the size of the fibers were observed. Sample preparation included liquid nitrogen immersion and breaking of scaffolds using gentle pestle hits, gold coating, and subsequently fixation of sample on aluminum stubs by using carbon tape. The images were collected through the equipment’s software. To determine the average strand and pore diameters (Feret’s diameter) from SEM images, ImageJ 1.54d software (National Institutes of Health, Bethesda, MD, USA) was used.

#### 2.3.4. Mechanical Properties

All tests were performed on cylindrical samples at room temperature using the Shimadzu Autograph AGS-X 20kN (Shimadzu, Tokyo, Japan) testing machine. The diameter and thickness of all samples were measured before testing. Testing was conducted at a constant loading rate of 0.5 mm/min and a maximum loading of 4000 N.

#### 2.3.5. In Vitro Chemical Bioactivity

Bioactivity was evaluated after immersing the scaffolds in simulated body fluid (SBF) solution at 37 °C for 28 days with the help of SEM coupled with EDX spectroscopy, as well as through measuring throughout the experiment the weight of the scaffolds, the pH, and conductivity of the SBF. An inoLab Multi 9630 IDS (Xylem, Washingtonc, DC, USA) pH meter was used. The preparation of the SBF solution was performed according to Kokubo’s recipe and procedure [29]. The SBF was not refreshed during the experiment. After immersion, the scaffolds were removed from the SBF medium, rinsed with deionized water, and dried at room temperature. The samples were analyzed using SEM and energy-dispersive X-ray (EDX) to determine the formation of the apatite layer on the surface of the scaffolds after 28 days.

#### 2.3.6. In Vitro Biological Evaluation

Mouse pre-osteoblasts (MC3T3-E1 cell line) were grown in Dulbecco Modified Eagle’s Medium (Invitrogen, Waltham, MA, USA) with 10% fetal bovine serum (Gibco, Waltham, MA, USA) at 37 °C in a humidified atmosphere with 5% CO_2_. The cells were seeded at a cell density of 6 × 10^4^ cells/cm^2^ on the tissue culture plastic surface (TCPS) which served as a control, on the top of the PCL 70% (wt.)–HA/SrHA 30% (wt.) scaffolds which were previously sterilized under UV light for 2 h. After 24 h of incubation in standard conditions, the biocompatibility tests were performed.

The cellular viability was measured using the 3-(4,5-dimethylthiazol-2-yl)-2,5-diphenyltetrazolium bromide (Sigma-Aldrich, St. Louis, MO, USA) assay (MTT). The culture medium was removed at the end of incubation time and the cells were incubated with 1 mg/mL MTT solution for 2 h at 37 °C. The purple formazan crystals formed in the viable cells were dissolved with 2-propanol (Sigma-Aldrich, St. Louis, MO, USA) and the absorbance was measured at 595 nm using a microplate reader (FlexStation 3, Molecular Devices, Sunnyvale, CA, USA).

The concentration of nitric oxide (NO) in the culture medium collected after the 24 h of incubation was measured using the Griess reagent, a stoichiometric solution (*v*/*v*) of 0.1% naphthylethylendiamine dihydrochloride, 1% sulfanilamide in 5% phosphoric acid. The absorbance of the mix formed by equal volumes of medium supernatants and Griess reagent was read at 550 nm using a microplate reader and the NO concentration was calculated from the NaNO_2_ standard curve.

The culture medium was harvested after 24 h of osteoblasts’ growth in the presence of tested samples and used for lactate dehydrogenase (LDH) release measurement with Cytotoxicity Detection KitPLUS (Roche, Indianapolis, IN, USA), following the manufacturer’s instructions. Volumes of 100 µL culture supernatants were mixed with 100 µL mix of catalyst and dye solution and incubated for 20 min in a dark place. After the reaction was stopped, the absorbance was read at 490 nm using a microplate reader (Flex Station 3, Molecular Devices, Sunnyvale, CA, USA).

Following incubation, the cells were fixed with 4% paraformaldehyde for 20 min and permeabilized with 0.1% Triton X-100 2% bovine serum albumin for 45 min. The actin filaments were stained with 10 µg/mL phalloidin-FITC (fluorescein isothiocyanate) and the nuclei were counterstained with 2 µg/mL DAPI (4′,6-diamino-2-phenylindole). An Olympus IX71 inverted fluorescence microscope was used to capture the images.

The in vitro assays were performed in triplicates and the results were calculated as mean ± standard deviation (SD) of three independent experiments. The statistical analysis was carried out on three replicates per sample by the unpaired Student t-test, and differences were considered significant for a *p*-value less than 0.05.

#### 2.3.7. Statistical Analysis

Data are represented as mean ± standard deviation (S.D.). The graphs and statistical analysis were performed using MS Excel software for Microsoft 365 (Version 2404). Data were compared using one-way analysis of variance (ANOVA), followed by a two tails *t*-test. Values of *p* < 0.05 were considered statistically significant.

## 3. Results and Discussion

### 3.1. PCL 70% (wt.)–HA/SrHA 30% (wt.) Scaffolds Design

**Figure 1:**
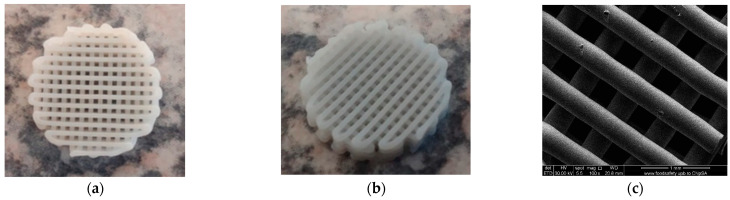
PCL 70% (wt.)–SrHA 30% (wt.) scaffolds obtained using HAHTSr10% powder: (**a**) macroscopic top-view, (**b**) macroscopic lateral-view, (**c**) top-view SEM images in EDT mode.

The macro-morphology of PCL, PCL-HA, and PCL–SrHA scaffolds (Figure 1a,b) indicate good compliance with the CAD model. The scaffolds exhibit a regular macroscopic porous structure and are stacked layer by layer. The strands are well defined and clearly observed both macroscopically and microscopically (Figure 1c). There was no obvious difference between the scaffolds.

**Figure 2:**
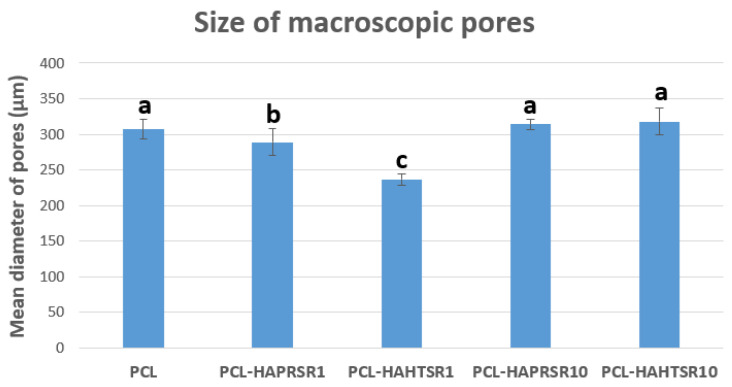
Macropore diameter measurements on PCL and 70% PCL–30% SrHA composites. Results are represented as mean ± SD; different letters indicate significant differences between each sample (*p* < 0.05).

A higher specific surface area has a direct relationship with the increase in the adsorption of macromolecules and proteins, particularly involved with the induction of osteogenesis. The diameter of interconnected pores is recommended to be at least 300 μm to allow proper cell migration inside the grafts and proper angiogenesis, which are two of the most important aspects for new-bone formation inside existing pores [30]. Macropores of the obtained scaffolds were measured from the SEM micrographs using ImageJ 1.54d software (Figure 2) and the obtained results follow the recommendations found in the literature, with mean diameter of pores varying between 230–320 μm. The results are similar, with statistically significant differences only for the samples with 1%Sr.

### 3.2. Thermogravimetric (TG) Analysis

**Figure 3:**
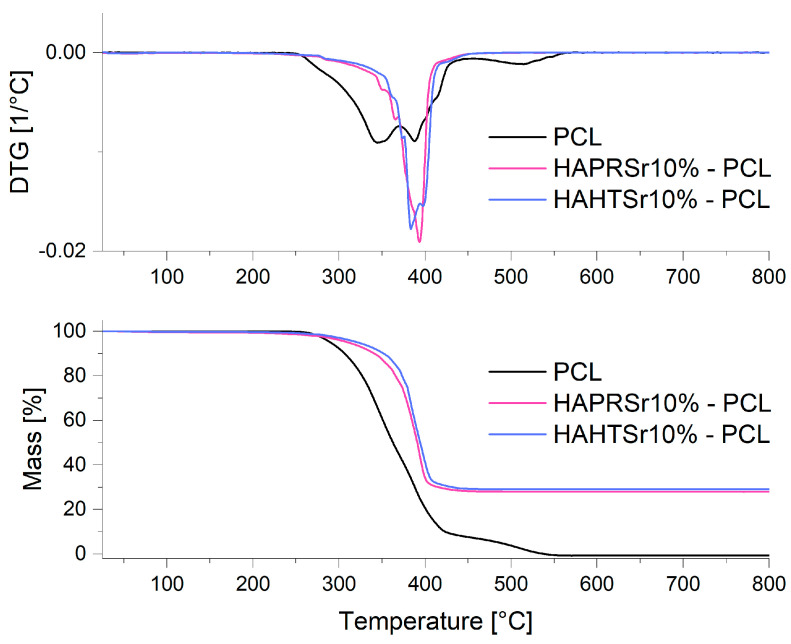
TG/DTG measurements on PCL and composite scaffolds containing HAPRSr10% and HAHTSr10%.

TG measurements were conducted on the 3D-printed scaffolds to quantify the HA/SrHA content at the end of the scaffold fabrication process. A sample of ~12 mg in weight was heated to 800 °C at a rate of 10 °C/min, and the sample weight loss was recorded over time. In addition, for control, a PCL pellet (~15 mg in weight) was tested in the same way. TG analysis was used to assess the composition (wt%) of the composite materials prepared through the melt-blending technique, as well as their thermal stability. All composite materials had experimental compositions close to the design value of 70% wt. PCL and 30% wt. HA/SrHA, as seen in Table 1. All the scaffolds demonstrated a one-step decomposition profile with a single transition temperature and similar thermal stability, with decomposition starting around 250 °C; however, the scaffolds printed with HA/SrHA material appeared to finish degradation at a lower temperature compared to the simple PCL scaffolds, as seen in Figure 3, for composites containing HAPRSr10% and HASR10%. This may be due to a catalytic effect of these powders and most likely a better thermal conductivity within the mass.

Considering the fact that ceramic component HA/SrHA is the only component that does not decompose during the TGA process, it can be assumed that during the entire processing, no heterogeneities are obtained, and the final composition is close to the desired one, for all the samples obtained with precipitated or hydrothermal HA/SrHA.

### 3.3. Scanning Electron Microscopy

**Figure 4:**
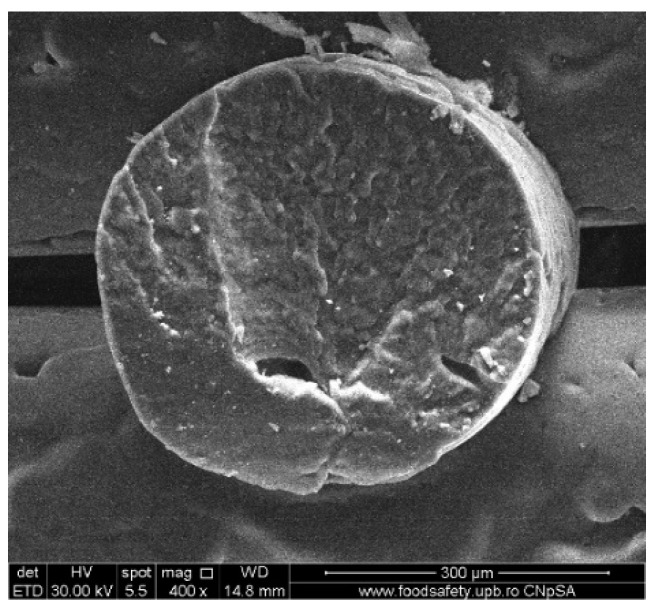
Cross-section SEM images of 100% PCL scaffolds.

**Figure 5:**
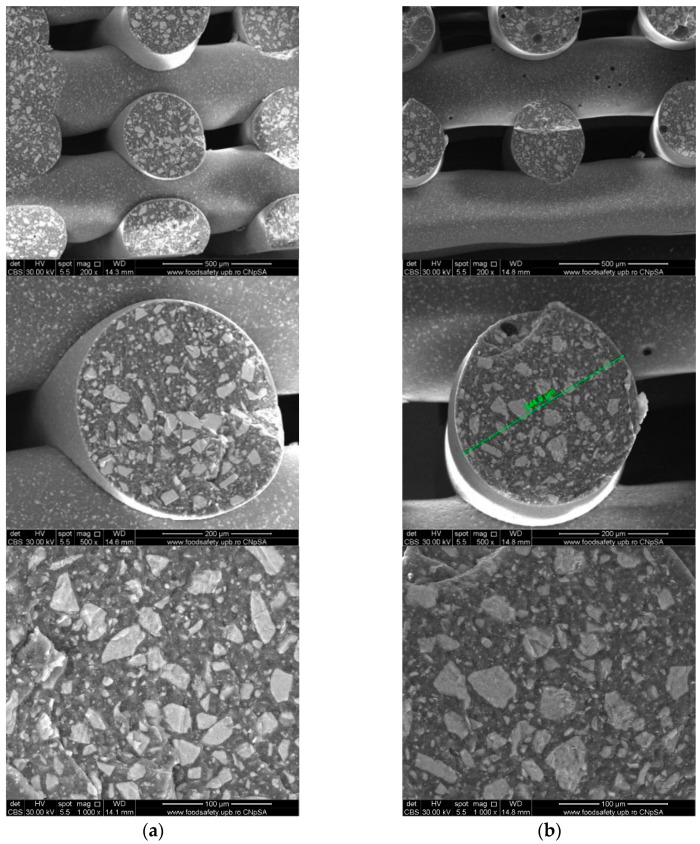
Cross-section SEM images of 70% PCL–30% SrHA composite scaffolds (**a**) PCL–HAPR-Sr1%, (**b**) PCL–HAPR-Sr10%.

**Figure 6:**
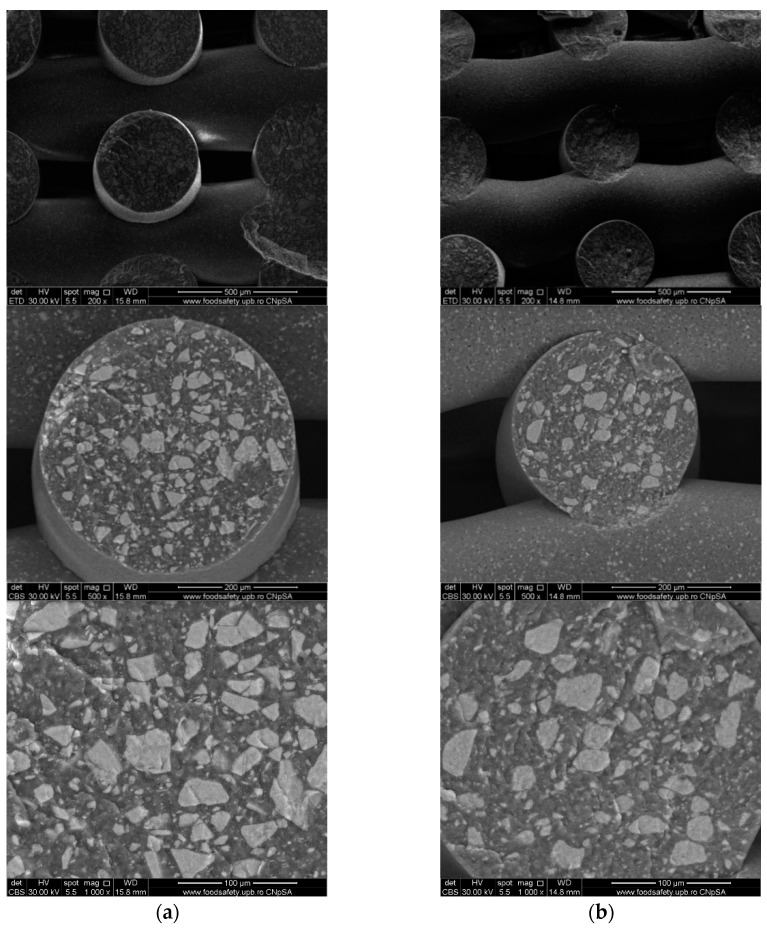
SEM images of 70% PCL–30% SrHA composite scaffolds (**a**) PCL–HAHT-Sr1%, (**b**) PCL–HAHT-Sr10%.

**Figure 7:**
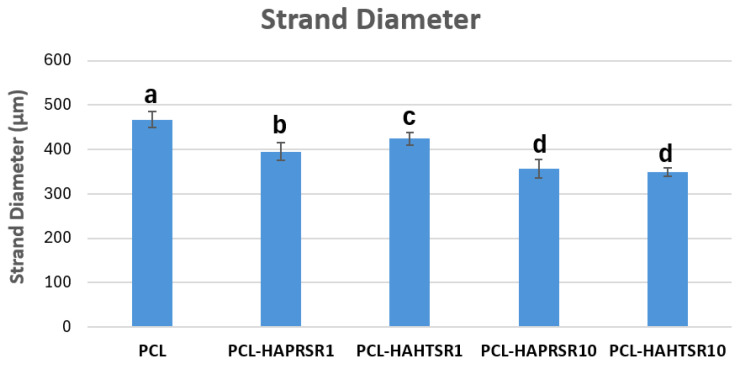
Strand diameter measurements on PCL and 70% PCL–30% SrHA composites. Results are represented as mean ± SD; different letters indicate significant differences between each sample (*p* < 0.005).

To investigate the obtained scaffolds’ surface morphology and strands’ dimensions, SEM analysis was performed. Morphological evaluation of the 3D-printed scaffolds at different magnifications showing strands and surfaces indicate that the printed scaffolds displayed a high level of fidelity to the original CAD model (Figure 4, Figure 5 and Figure 6), with completely open and interconnected pore architecture. Strand diameter was measured using ImageJ 1.54d software for several samples (Figure 7) and the average value was close to the theoretical target value of the printing needle (400 μm). Simple PCL strands had statistically significant bigger values than the composite strands due to the typical behavior of viscoelastic polymer-based biomaterial inks, that tend to expand upon extrusion from the nozzle [31]. However, no significant differences were observed between the composites containing 10%Sr.

**Figure 8:**
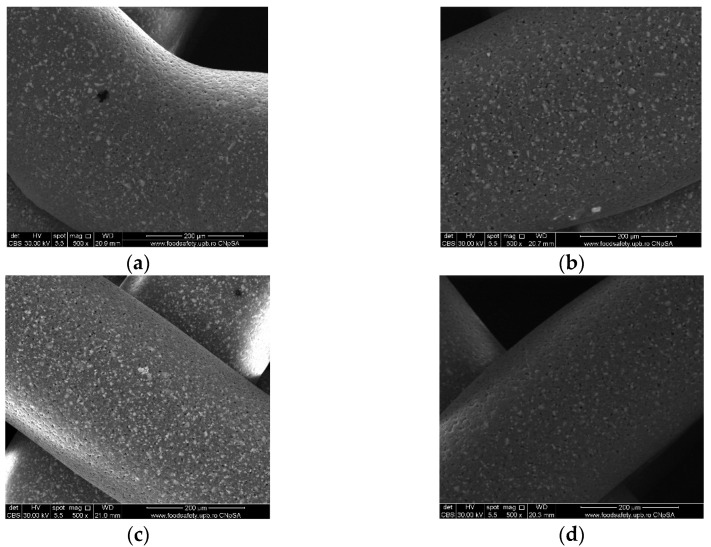
Top-view SEM images of 70% PCL–30% SrHA composite scaffolds (**a**) PCL–HAPR-Sr1%, (**b**) PCL–HAHT-Sr1%, (**c**) PCL–HAPR-Sr10%, (**d**) PCL–HAHT-Sr10%.

Images acquired at higher magnification using a top-view scan and the backscattered electron (CBS) mode evidenced the presence of SrHA particles and micro-porosity on the surface of all analyzed samples (Figure 8). Composite filaments’ surface appeared less smooth than the simple PCL. More and smaller micropores can be observed in the case of composite scaffolds, compared with simple PCL scaffolds. This might be due to the collision of ceramic particles with PCL crystalline regions growing during solidification, as other authors mention [32]. Cross-section images, even on lower magnification, indicate the agglomeration or clumping of the ceramic particles in the composite-based samples in both CBS and Everhart–Thornley Detector (ETD) modes (Figure 5 and Figure 6) but there is no critical agglomeration which means that homogenous materials were obtained.

**Figure 9:**
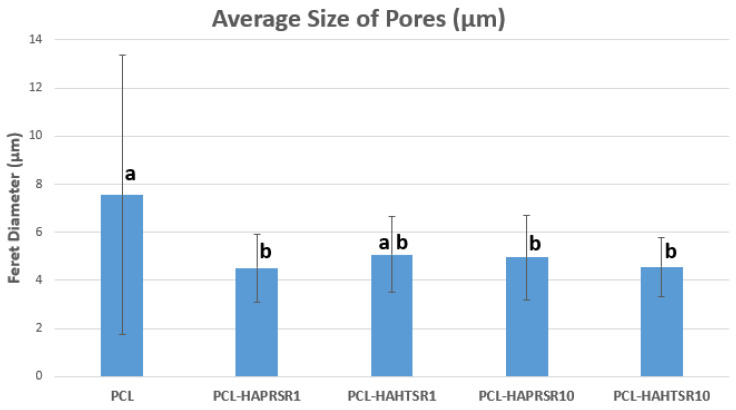
The average size of pores using Feret diameter measurements on PCL and 70% PCL–30% SrHA composite samples. Results are represented as mean ± SD; different letters indicate significant differences between each sample (*p* < 0.05).

The surface of the samples (top-view) is very important because this is in direct contact with the surrounding fluids, cells, and tissues. The surfaces reveal HA particles partially embedded into the polymer matrix but also micrometric pores. The surface roughness is important because it can allow a better cell–graft interaction and thus a better cell attachment onto the surface. Pores were analyzed quantitatively by ImageJ and presented using average Feret diameter (Figure 9). The average size was similar in all composite samples, with no statistically significant differences, but statistically different from the PCL sample that presented pore shape polydispersity and a higher average diameter. These measurements indicate that the observed pores are in the optimal diameter range for cell adhesion (1–10 μm in diameter), similar to the microstructure of tissue- and organ-derived decellularized extracellular matrices [33]. Surface pores of 3–12 μm size are important for direct cell–cell contacts, migration, and/or invasion [34]. Together with the larger macropores intentionally obtained via CAD design, these smaller pores create a multiscale pore architecture that substantially promotes cell adhesion, viability, and proliferation.

### 3.4. Mechanical Characterization

**Figure 10:**
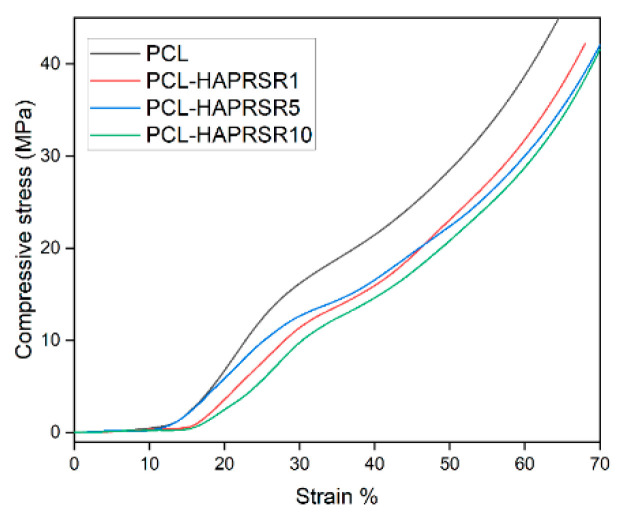
Compressive stress–strain curves for PCL, PCL–HAPR-Sr1%, PCL–HAPR-Sr5%, PCL–HAPR-Sr10% scaffolds.

**Figure 11:**
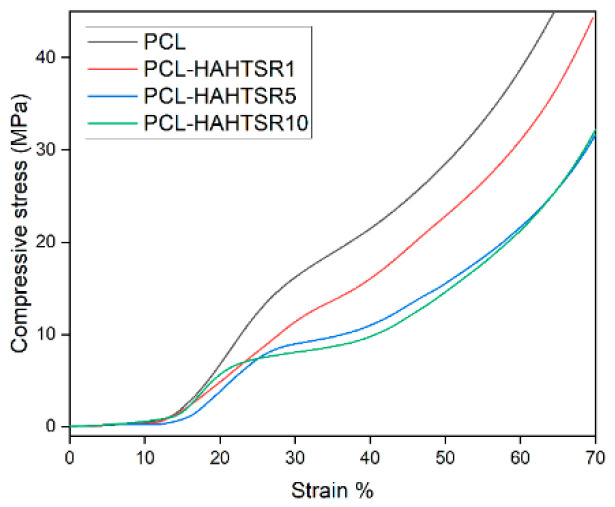
Compressive stress–strain curves for PCL, PCL–HAPR-Sr1%, PCL–HAPR-Sr5%, PCL–HAPR-Sr10% scaffolds.

The compressive mechanical properties of simple PCL and PCL–SrHA composite scaffolds were investigated, as they represent key requirements for bone scaffolds. The representative compressive stress–strain curves are shown in Figure 10 and Figure 11. Stress–strain curves of both simple PCL and PCL–SrHA composite scaffolds include the three distinct regions also reported by other authors [35], respectively: firstly, the linear elastic region, secondly, the plateau of roughly constant stress, and thirdly, the final densification region of steeply rising stress. Other authors [36] indicate that these three main regions correspond, firstly, to the increase in compression force that leads to an increase in strength, when the thin bonding bridges between the struts begin to crack, secondly, to the strength decrease due to the struts cracking in the stress accumulation areas, and thirdly, strength increases again due to the rearrangement of the material.

PCL–SrHA composite scaffolds have similar mechanical properties to that of simple PCL scaffolds. PR-obtained SrHA displayed stress–strain curves with very similar trends, thus indicating reproducible mechanical results of the manufacturing process. While comparing the two groups of samples, it seems that composites containing PR-obtained SrHA perform better. Mechanical performances need to match those of the host bone tissue, especially in the load-bearing implants [31], and should have a minimum compressive strength of 2 MPa, a requirement that all tested scaffolds meet [36]. Other authors mention a possible optimal range of the compressive strength between 2 to 5 Mpa for cancellous bone, 5 and 131 Mpa for alveolar bone, and 131 to 224 Mpa for compact bone [37]. We took in consideration for this comparison the second inflection point of the stress–strain curve, corresponding to the collapse of material and clogging of the scaffold pores [38]. The compressive stress of pure PCL scaffold is slightly higher than the compressive stress of PCL–SrHA composite scaffolds, as shown in Figure 10 and Figure 11. The outcome might be the consequence of the poor adhesion at the interface between the two different components due to the mechanical mixing and lack of chemical blending [32].

### 3.5. Chemical In Vitro Bioactivity

The ability to form new apatite layer on the surface of the PCL 70% (wt.)–HA/SrHA 30% (wt.) scaffolds was investigated by immersing them in SBF solution (pH 7.4) at 37 °C for 28 days. The weight of the scaffolds, the pH value, and conductivity of SBF solutions containing the scaffolds were measured at several immersion periods, up to 28 days. 

The formation of an apatitic layer is critical for osteoblast cell adhesion and proliferation. Upon contact with SBF, the scaffold undergoes an ionic exchange with its surrounding medium [39]. The dry weight of the scaffolds was measured before and after the immersion in SBF and the percentage of weight increase was calculated as follows:

(1) $$W_{P}={(W}_{F}{-W}_{I})/\;W_{I}\times 100\%$$

where *W_P_* represents the percentage of weight increase, *W_F_* is the weight of sample after immersion in SBF, and *W_I_* before immersion in SBF.

**Figure 12:**
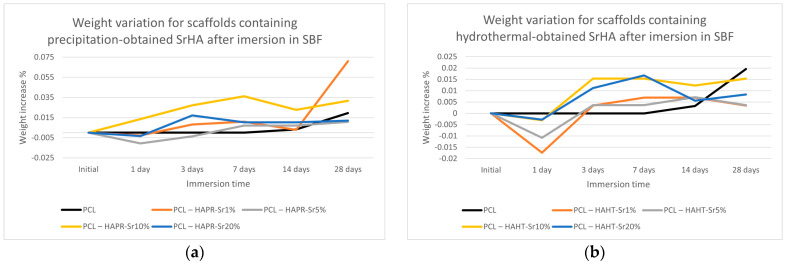
Weight variation for scaffolds containing precipitation- (**a**) and hydrothermal- (**b**) obtained SrHA after immersion in SBF.

As shown in Figure 12, the weight of the scaffolds varied only slightly, but in a similar fashion for most of the samples. Scaffolds containing precipitation-obtained SrHA had a higher increase in weight in general, with PCL–HAPR-Sr1% and PCL–HAPR-Sr10% having noticeable variations. Pristine PCL scaffolds are considered to have an initially very low erosion of less than 1 wt.% in the first month of exposure to physiological conditions. In contrast, composite scaffolds are considered to exhibit weight loss within the first week of exposure to physiological conditions [37]. Possible initial degradation may have happened to the composite scaffold in contrast with the pristine PCL scaffolds (Figure 12). In the case of the PCL-HAHT samples, this possible event is emphasized by a more dramatic weight loss (Figure 12b). Towards the later stages of the experiment, the apatite layer deposition may have reversed the loss of weight.

**Figure 13:**
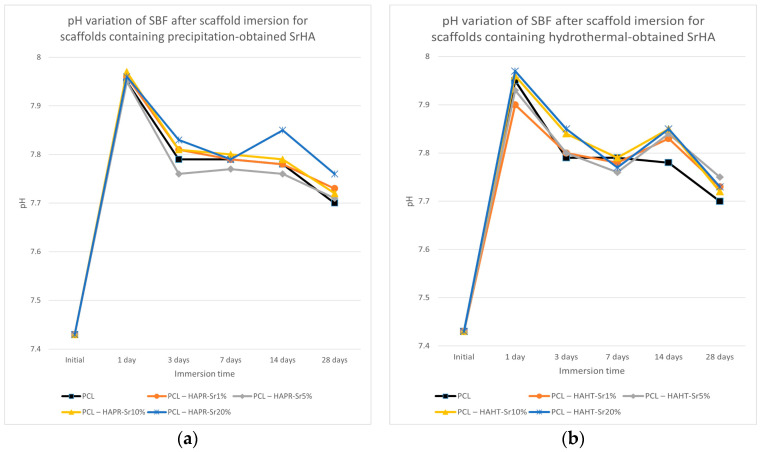
pH variation of SBF after scaffold containing precipitation- (**a**) and hydrothermal- (**b**) obtained SrHA immersion for various periods of time.

The pH of the SBF solution containing the scaffolds overall increased (Figure 13). The partial dissolution of the SrHA surface layers may be the cause of the initial increase in pH, and the subsequent formation of apatite with the consumption of OH^-^ ions may cause the decrease in pH [40]. Slightly lower values were observed for pristine PCL scaffolds. This effect may contribute to ameliorating the inflammation caused by the acidic microenvironment usually produced by degradation products of PCL [37]. Both the increase in degradation of the composite scaffolds compared to simple PCL scaffolds and the increase in pH are advantages that confirm the necessity to incorporate HA/SrHA into PCL scaffolds.

**Figure 14:**
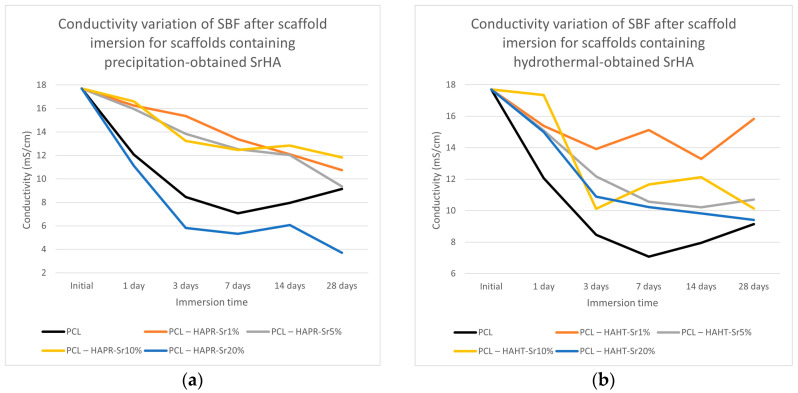
Conductivity variation of SBF after scaffold containing precipitation- (**a**) and hydrothermal- (**b**) obtained SrHA immersion for various periods of time.

The conductivity measurements of SBF after scaffold immersion, presented in Figure 14, show discontinuous variation, probably due to the simultaneous solubilization and mineralization processes taking place at different rates [36]. The overall lower conductivity values may be related to the reduction of Ca^2+^ and PO_4_^3−^ concentrations in SBF compared with the values of the initial solution, due to the formation of an apatitic layer [38,41].

**Figure 15:**
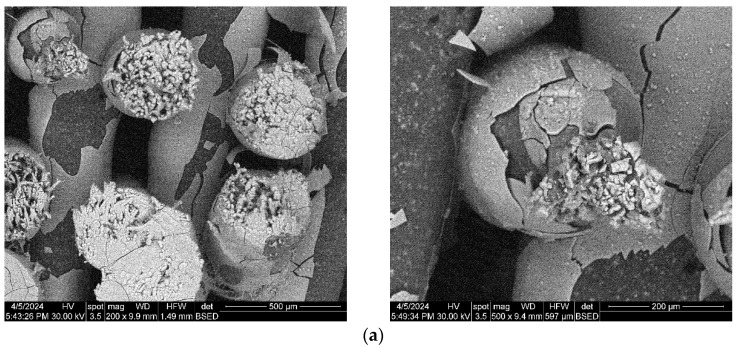

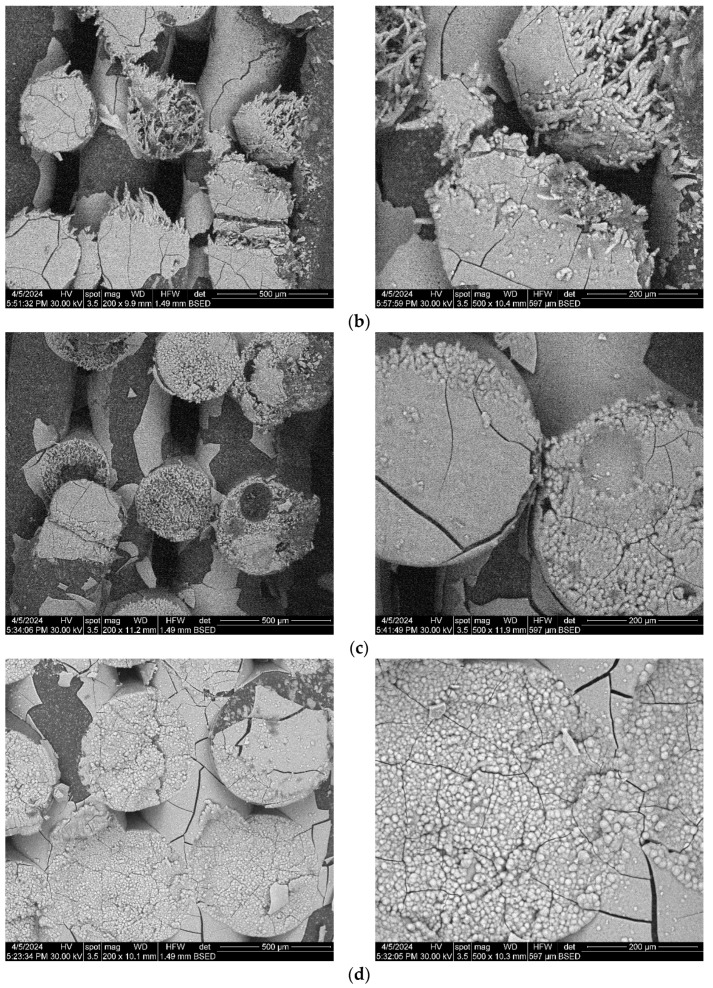
SEM images of the 3D-printed composite scaffolds containing precipitation-obtained SrHA after 28 days of immersion in SBF: (**a**) PCL-HAPR-Sr1%, (**b**) PCL-HAPR-Sr5%, (**c**) PCL-HAPR-Sr10%, (**d**) PCL-HAPR-Sr20%.

**Figure 16:**
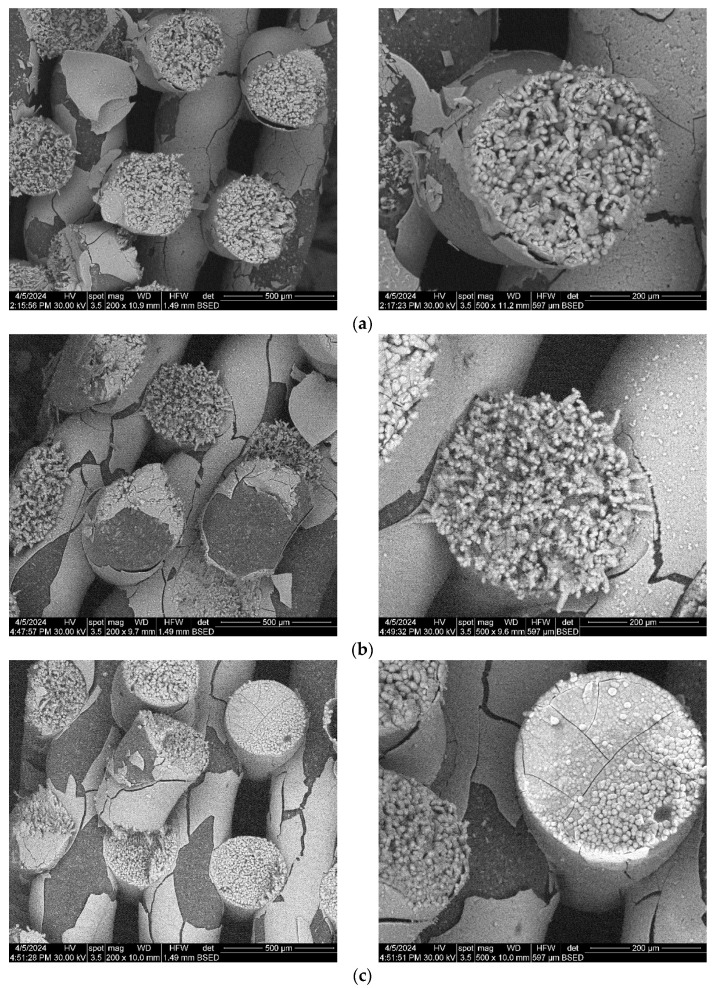

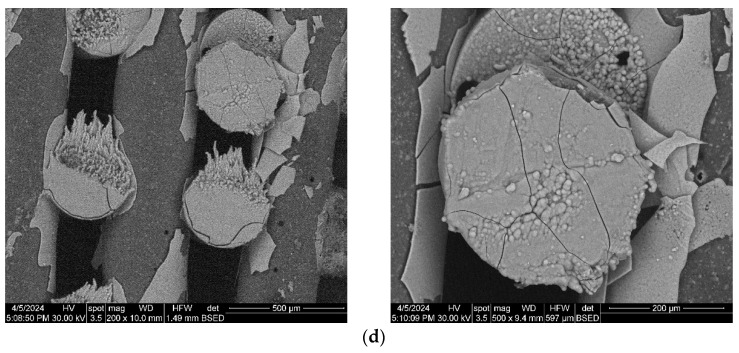
SEM images of the 3D-printed composite scaffolds containing hydrothermal-obtained SrHA after 28 days of immersion in SBF: (**a**) PCL-HAHT-Sr1%, (**b**) PCL-HAHT-Sr5%, (**c**) PCL-HAHT-Sr10%, (**d**) PCL-HAHT-Sr20%.

The composite scaffolds’ morphology was assessed post-28-day immersion in simulated body fluid (SBF) utilizing scanning electron microscopy (SEM), with the findings depicted in Figure 15 and Figure 16. Prior to immersion, surface features like pores and roughness remained unchanged by the SBF. However, a continuous apatite coating, approximately 6 µm thick, was detected on all scaffolds, supported by EDS spectra and mapping (Figure S1, Supporting Information). Notably, a tendency for cracking or exfoliation of the apatite layer was observed across all samples, indicating potential inadequate adhesion between the scaffold surface (particularly PCL) and the mineral coating. Factors such as pH variations, ion exchange, and scaffold component dissolution (HA) may influence the stability and integrity of the mineral coating. Cross-section SEM images revealed apatite-filled pores within the scaffold and the formation of micronic agglomerates. These results imply the scaffolds’ effectiveness in promoting mineralization, yet underscore the need for further optimization of fabrication protocols and material design strategies to improve adhesion and durability of the apatite layer on 3D-printed scaffolds.

### 3.6. Biological In Vitro Scaffold Performance

**Figure 17:**
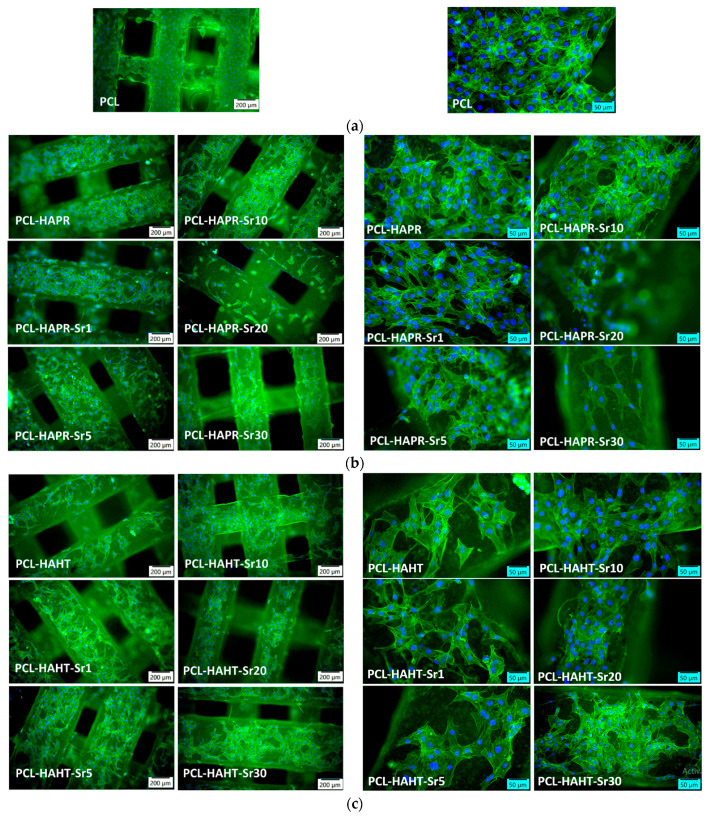
Representative images of fluorescence microscopy with different magnifications after 24 h of incubation showing the actin cytoskeleton staining in MC3T3-E1 pre-osteoblasts attached on the surface of 3D-printed scaffolds: (**a**) containing only PCL, (**b**) containing precipitation-obtained SrHA (**c**) containing hydrothermal-obtained SrHA. F-actin was stained in green with phalloidin-FITC and nuclei in blue with DAPI.

In order to assess the biocompatibility of the different types of 3D-printed scaffolds, the F-actin staining of MC3T3-E1 cells attached on the surface of these materials was performed. The images shown in Figure 17 revealed a good adhesion of these cells for all types of scaffolds tested, being distributed within the whole surface of them after 24 h of incubation. A good cytocompatibility was noticed for PCL sample (Figure 17a), as it was previously reported [27]. In the case of samples containing SrHA obtained by precipitation, the best cell morphology was achieved for Sr1, 5, and 10 (Figure 17b). For HAHT-based samples, the addition of Sr increases the cell attachment compared to PCL-HAHT scaffold (Figure 17c). The analysis of images captured at a higher magnification indicated a higher cell density for PCL-HAPR, PCL-HAPR-Sr1, 5, and 10, being observed an organized actin filaments network with numerous cell-to-cell junctions, which suggest a functional osteoblast phenotype. In addition, a good cell interaction was shown for PCL-HAHT-Sr30.

**Figure 18:**
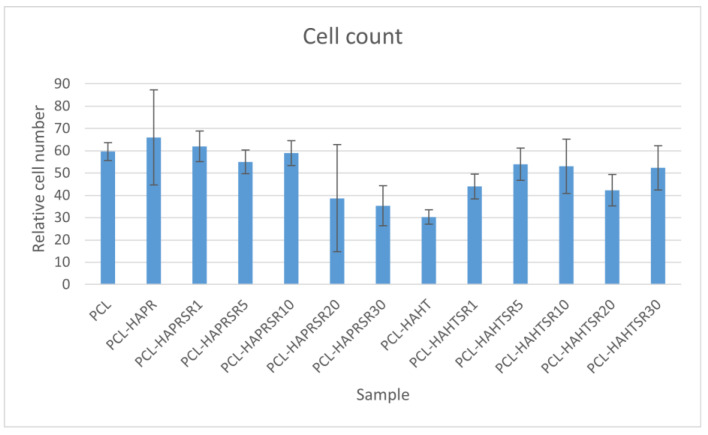
Quantitative analysis of cell adhesion on the scaffolds after 24 h of incubation. Results are represented as mean ± SD (*n* = 3).

Relative cell adhesion was investigated for all the samples using images of fluorescence microscopy. Images were analyzed quantitatively with the help of ImageJ and results were presented as average cell number (Figure 18). In the case of composites containing precipitation-obtained SrHA, results are higher for lower Sr concentrations, while in the case of hydrothermal-obtained samples, better results are obtained at higher concentrations, apart from PCL–HAHT-Sr20%. Although pristine PCL scaffolds also had good results, in confirming the biocompatible nature of this polymer and its suitability in developing new composite materials, it is often considered that the improvement brought by the added HA/SrHA is more visible on longer periods of time [27].

**Figure 19:**
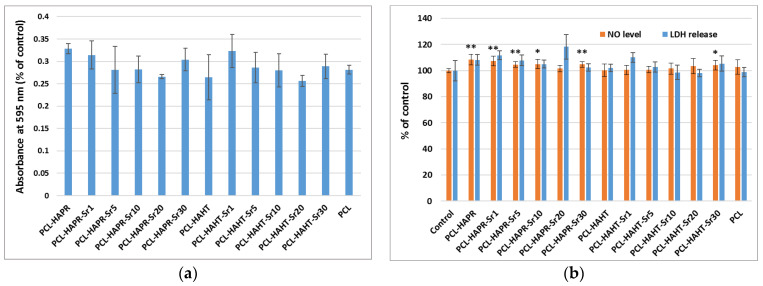
Biocompatibility evaluation of 3D-printed scaffolds, containing PCL and HA/SrHA obtained by precipitation and hydrothermal method, after 24 h of incubation with MC3T3-E1 pre-osteoblasts by measuring the viability of cells attached on these scaffolds: (**a**) cell viability was assessed by MTT assay and represented as mean ± SD (n = 2); (**b**) NO level and LDH release in culture medium. The results were calculated as mean ± SD (n = 3) and expressed relative to control (cells incubated without scaffolds) for NO and LDH assays (* *p* < 0.05 and ** *p* < 0.01 compared to control).

The good cytocompatibility of all scaffolds was confirmed by the metabolic activity of MC3T3-E1 osteoblasts measured by MTT assay (Figure 19a), manifesting no important differences between the samples. However, the lowest values were noticed in the case of Sr20, for both types of HA. Furthermore, the biological evaluation included the examination of the cells attached to the plastic surface of the culture dish, under the scaffold. The F-actin staining revealed a high attachment of pre-osteoblasts (Figure S3 from Supporting Information), not being disturbed by the materials, confirming their biocompatibility for the neighboring cells in the case of tissue engineering application. Moreover, the NO level was unchanged compared to control cells (not exposed to materials), suggesting that the scaffolds did not induce inflammation (Figure 19b). In the case of LDH release, the highest level was obtained after the incubation with PCL-HAPR-Sr20, being in agreement with the results of MTT assay (Figure 19a) and F-actin staining (Figure 17). In this case, the decreased attachment could be correlated with a loss in cell membrane integrity.

## 4. Conclusions

PCL 70% (wt.)–HA/SrHA 30% (wt.) composite materials were obtained through melt-blending and used for the 3D printing of scaffolds through the FDM technique. Melt-blending is simple, cost-efficient, and avoids cytotoxicity associated with solvent residuals. The FDM technique is simple to use, reliable, and capable of putting into practice complex designs. Three-dimensional scaffold design was suited for overall biological performance due to adequate strand arrangement, layer thickness, and porosity. Experimental results indicate that the obtained 3D scaffolds favor the adhesion and proliferation of pre-osteoblasts attached to these surfaces, avoiding drawbacks associated with poor integration of scaffolds due to their low interaction with cells.

Overall good results were obtained, although in the case of F-actin staining, the best cell morphology was achieved for lower Sr concentrations, and subsequently, in the case of LDH release, the highest level was obtained after the incubation with PCL-HAPR-Sr20, being in agreement with the results of MTT assay and F-actin staining. The decreased attachment could be correlated with a loss in cell membrane integrity. Based on cell viability, quantitative analysis of cell adhesion and mechanical properties, we consider that samples containing SrHA with a lower concentration of Sr are better suited for bone tissue applications. Also, comparing the overall results between the scaffolds containing HA/SrHA obtained via different synthesis methods, we consider that the precipitation method is recommended. The precipitation method has the additional advantages of being more simple, easy, less expensive, and as previously shown [23], a more amorphous structure and a carbonated composition, capable of integrating better in the host tissue due to similarities with the natural-occurring apatite.

The manufacturing strategy applied in this study may be used for the development of more complex and patient-specific substitutes, allowing the inclusion of additional bioactive substances into these composites that better match the patient-specific needs of the native bone tissue. It is expected that similar strategies can become widely used and, thus, evolve as a next-generation medical device for managing osteoporosis fractures or even other bone defects.

## Figures and Tables

**Table 1:** TG measurements on 70% PCL–30% HA/SrHA composites.

Sample Name	Powder Synthesis Method	Powder Sr/(Ca+Sr) Molar Ratio (%)	Determined Content in the Scaffold (% wt.)	
			HA/SrHA	PCL
PCL-HAPRSr1	Precipitation	1	28.7	71.3
PCL-HAPRSr5		5	29.2	70.8
PCL-HAPRSr10		10	28.7	71.3
PCL-HAPRSr20		20	28.4	71.6
PCL-HAPRSr30		30	27.3	72.7
PCL-HAHTSr1	Hydrothermal	1	29.1	70.9
PCL-HAHTSr5		5	28.8	71.2
PCL-HAHTSr10		10	29.4	70.6
PCL-HAHTSr20		20	28.7	71.3
PCL-HAHTSr30		30	28.8	71.2

## Data Availability

Data are contained within the article and the Supplementary Materials.

## Supplementary Materials

The following supporting information can be downloaded at: https://www.mdpi.com/article/10.3390/polym16111511/s1, Figure S1: EDS mapping and spectra of all composite scaffolds; Figure S2: Cross-section SEM images of PCL-HAHT-Sr5% composite scaffold highlighting the thickness of the apatite covering layer; Figure S3: Representative fluorescence images of F-actin staining in MC3T3-E1 osteoblasts attached on the surface of tissue culture dish, under the 3D-printed scaffolds, after 24 h of incubation (green: actin filaments, blue: nuclei, objective magnification 16×). The control without any scaffold was represented by cells grown on the plastic surface of culture dish.
