# Supplementary material for: Three-Dimensional-Printed Composite Scaffolds Containing Poly-ε-Caprolactone and Strontium-Doped Hydroxyapatite for Osteoporotic Bone Restoration
Source: Polymers (Basel). 2024 May 27;16(11):1511. doi: 10.3390/polym16111511 (PMC11174839; doi:10.3390/polym16111511)
Supplement: Supplementary file 1 [file polymers-16-01511-s001.zip › polymers-3009248-supplementary.pdf]

PCL-HAHT-Sr1%

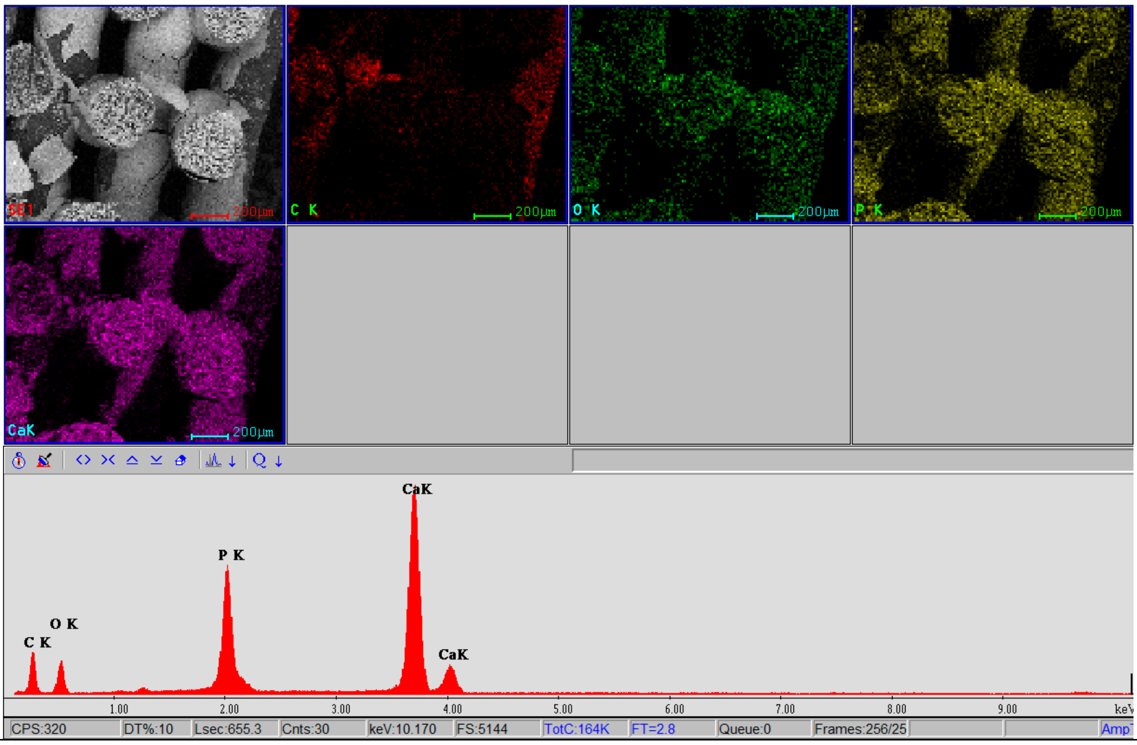

PCL-HAHT-Sr5%

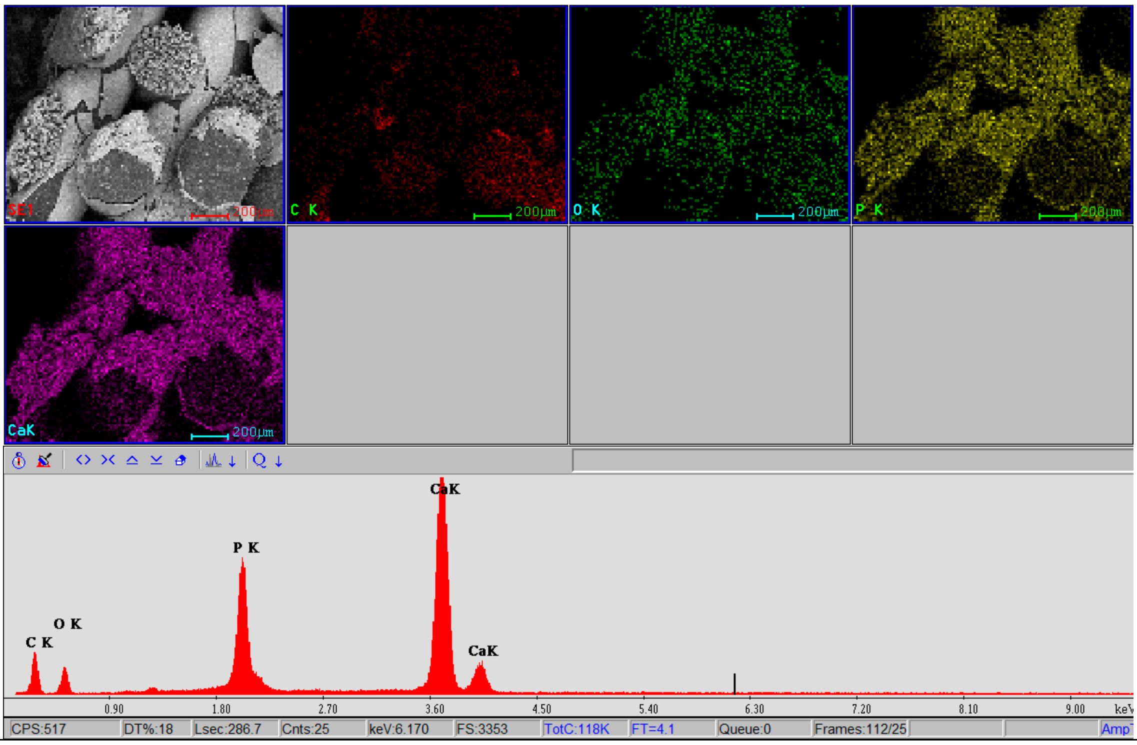

PCL-HAHT-Sr10%

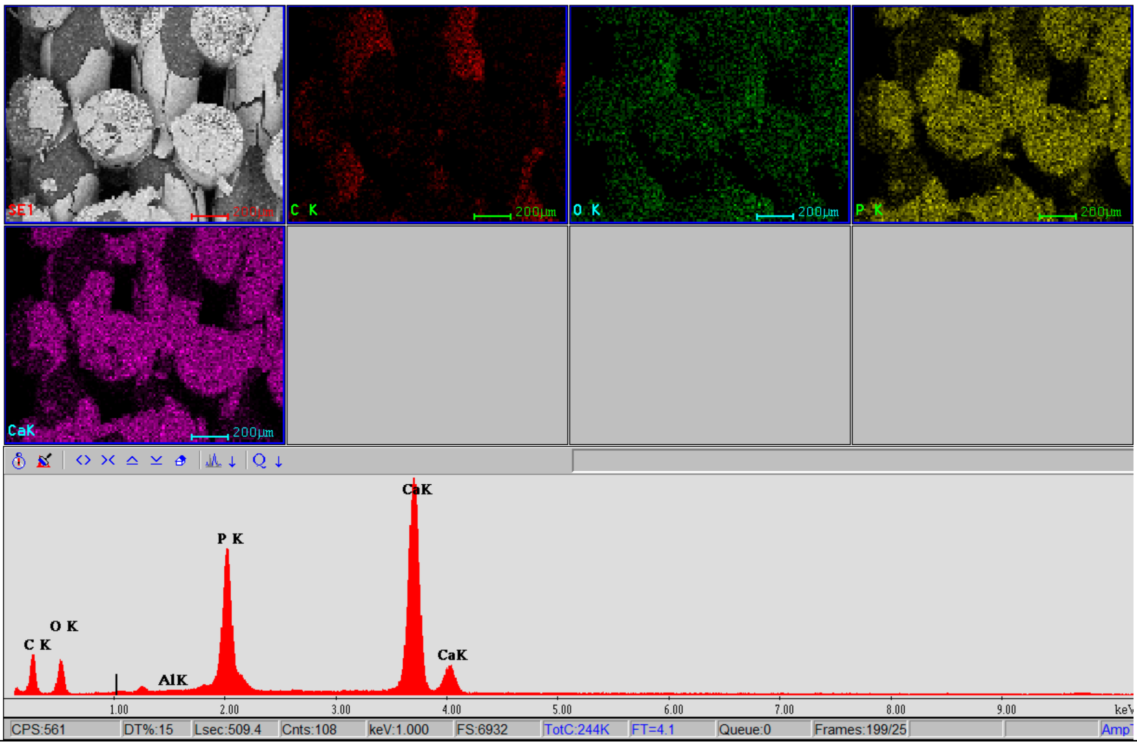

PCL-HAHT-Sr20%

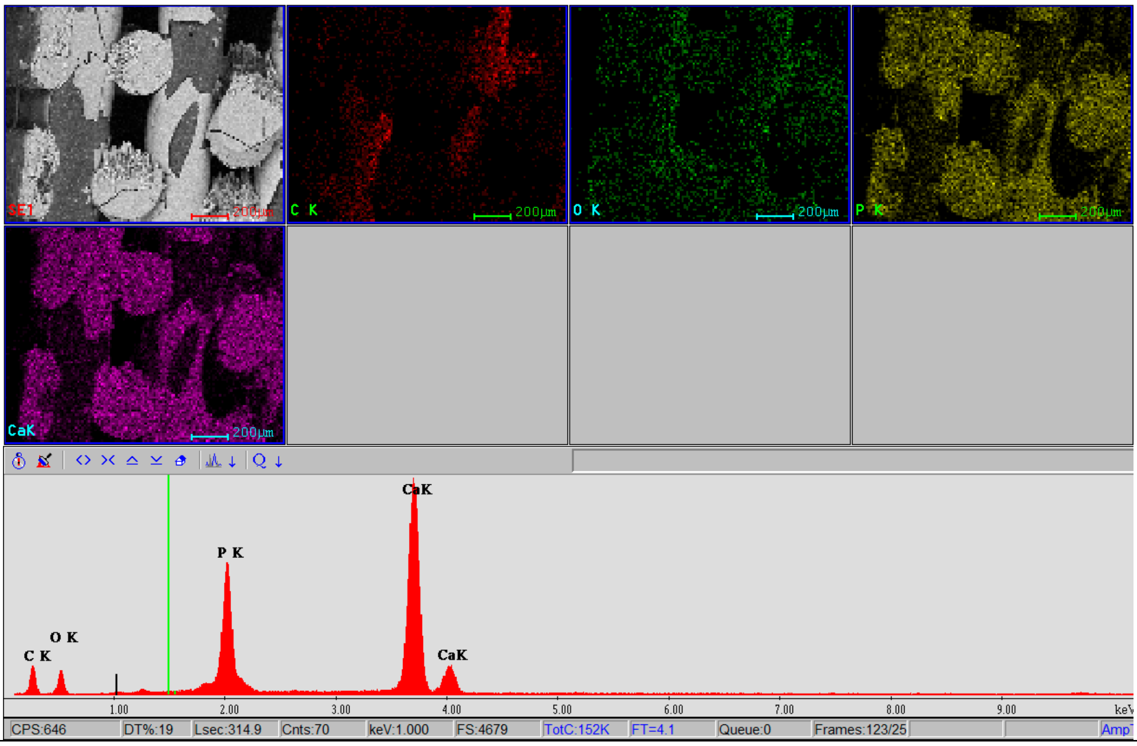

PCL-HAPR-Sr1%

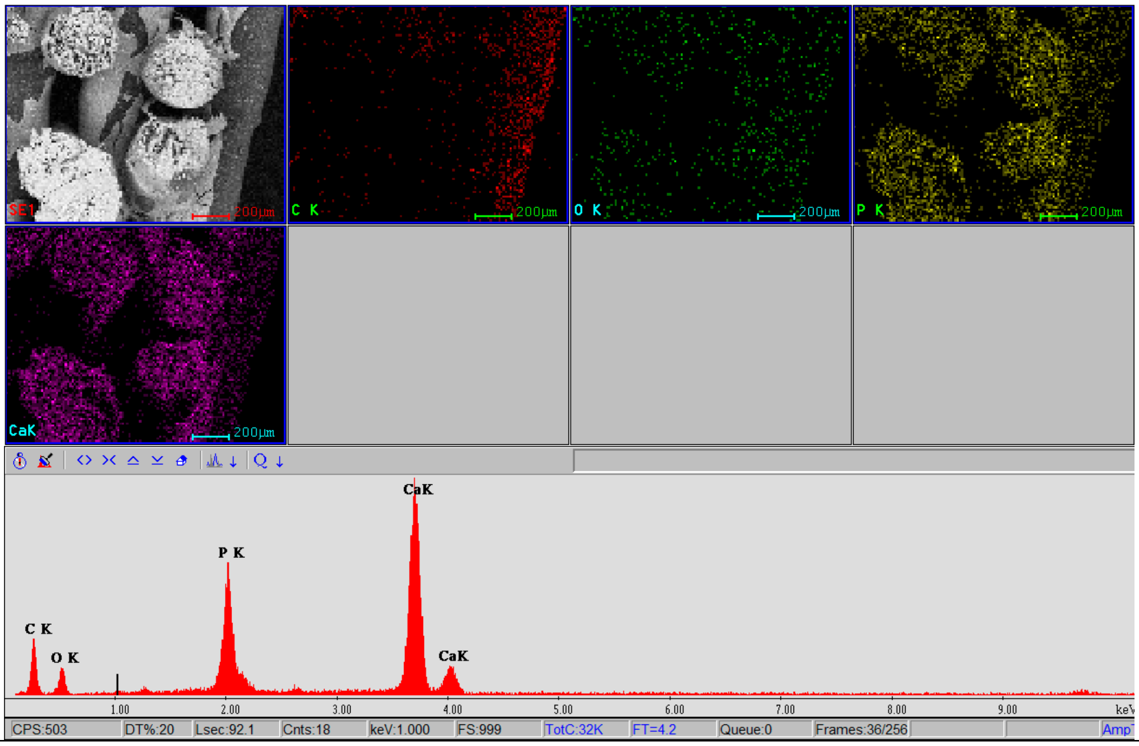

PCL-HAPR-Sr5%

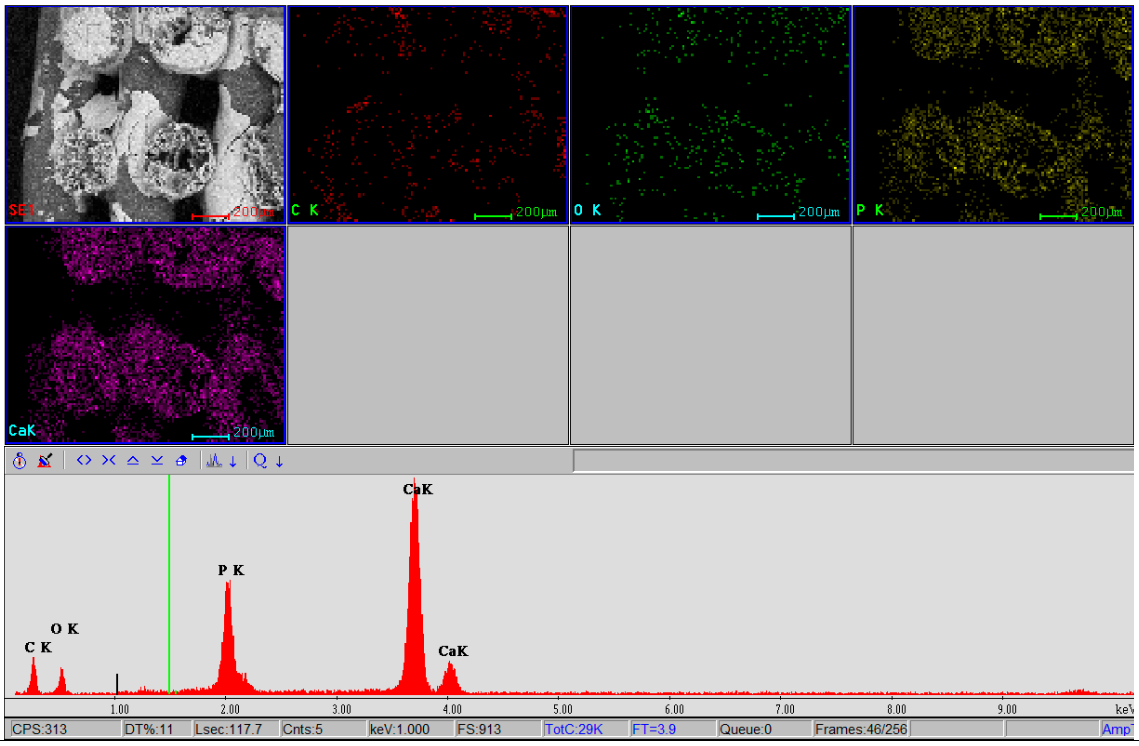

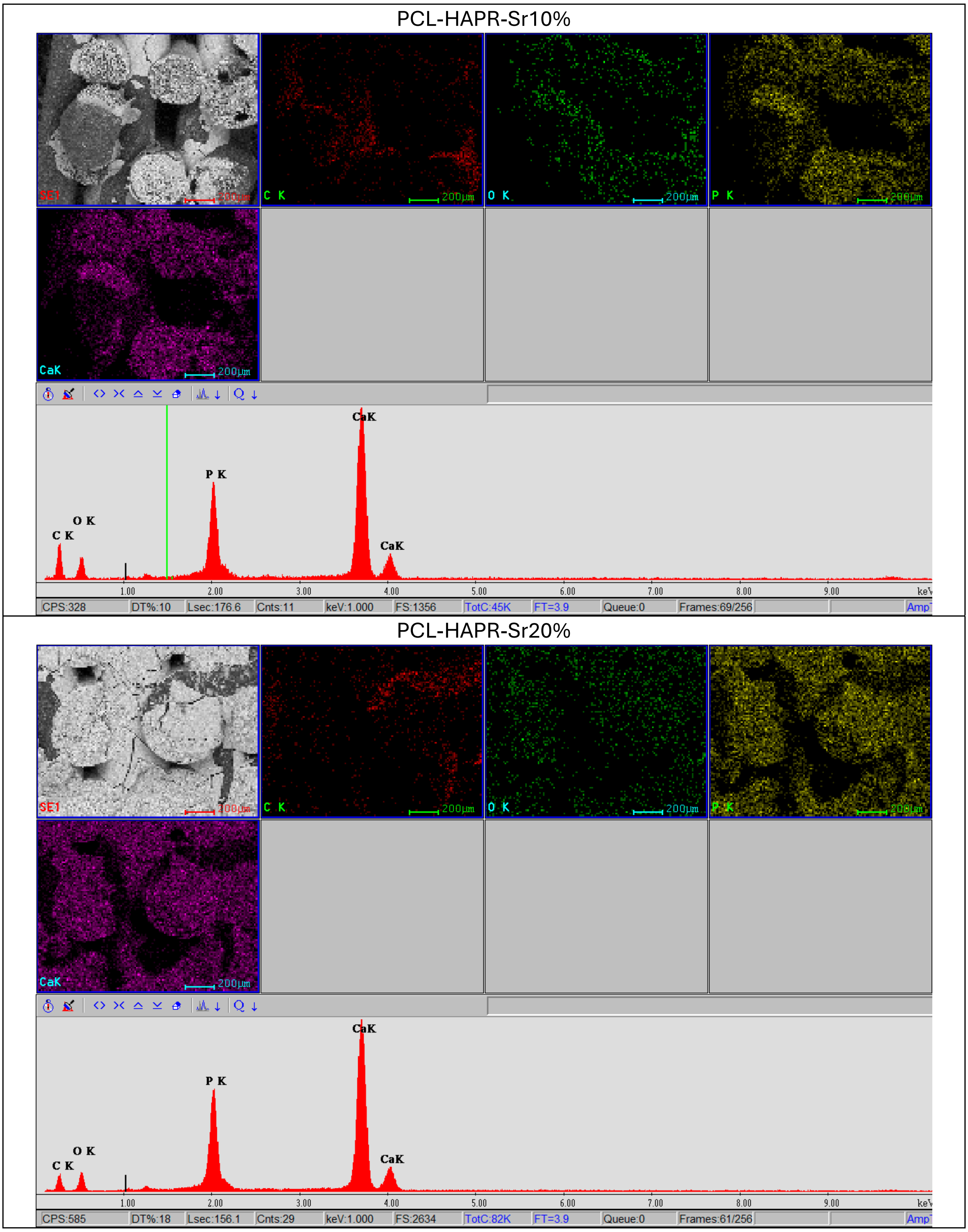

Figure S1. EDS mapping and spectra of all composite scaffolds

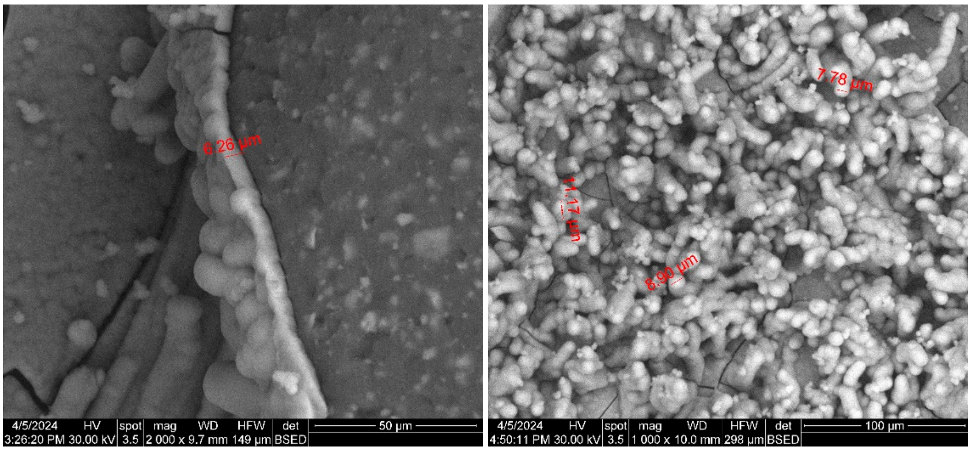

Figure S2. Cross-section SEM images of PCL-HAHT-Sr5% composite scaffold highlighting the thickness of the apatite covering layer

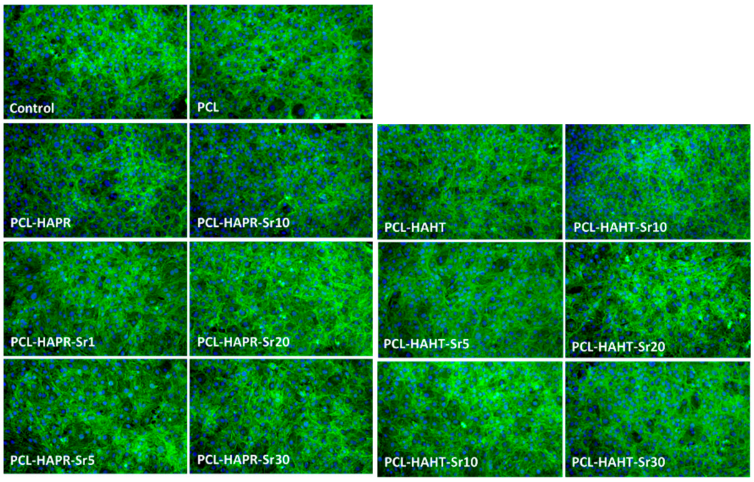

Figure S3. Representative fluorescence images of F-actin staining in MC3T3-E1 osteoblasts attached on the surface of tissue culture dish, under the 3D printed scaffolds, after 24 hours of incubation (green: actin filaments, blue: nuclei, objective magnification 16X). The control without any scaffold was represented by cells grown on the plastic surface of culture dish.
